# Manufacturing plant-based non-dairy and probiotic frozen desserts and their impact on physicochemical, sensory and functional aspects

**DOI:** 10.1007/s13197-024-05964-8

**Published:** 2024-03-10

**Authors:** Hayriye Akalın, Özer Kınık, Gülçin Şatır

**Affiliations:** 1 Department of Dairy Technology, Faculty of Agriculture, Ege University, İzmir, Turkey; 2 Department of Nutrition and Dietetics, Faculty of Health Sciences, Suleyman Demirel University, Isparta, Turkey

**Keywords:** Frozen dessert, Almond, Hazelnut, Lupin, Plant-based milk, *Lb. acidophilus*

## Abstract

The present study aimed to produce frozen dessert containing plant-based milk (almond, hazelnut, and lupine) and the probiotic *Lb. acidophilus* bacteria and to evaluate the chemical, microbiological and sensory properties during the 90 day-storage. Frozen dessert antioxidant capacity at day 0 and 90 of evaluation and changes in the phenolic compounds based on variations between different species were significant (*p* < 0.05). The differences in *Lb. acidophilus* counts between storage days were significant and values ranged from 4.15–8.99 log CFU/mL on the first day of storage to 3.61–7.06 at the end of the storage. Regarding the results of general acceptability in sensory evaluation, the highest color, taste and aroma scores was determined on day 0 in the hazelnut-lupine milk frozen dessert sample whereas the lowest was determined on day 30 in the almond-lupine milk frozen dessert sample. The samples with the highest antioxidant capacity were found on day 90 day in lupine frozen dessert (87.28 ± 0.007 mM) whereas the samples with the lowest antioxidant capacity were found on day 0 in the almond-hazelnut-lupine frozen dessert (18.83 ± 4.56 mM). Plant-based milk is considered suitable for the main ingredients in ice cream production, due to its health benefits its potential to be consumed as frozen dessert.

**Supplementary Information:**

The online version contains supplementary material available at 10.1007/s13197-024-05964-8.

## Introduction

Food is not only essential for life, but they also have a variety of good and bad environmental effects. As the highly industrialized and globalized food sector seeks to meet the expanding food demands of a growing population, it is estimated that the food industry contributes 26% of world greenhouse gas emissions (Kot et al. 2021). Taking the environmental variables into account, restricting the consumption of animal products is the most effective strategy, even if they play a significant role in our daily diet. Due to growing ethical concerns about animal welfare worldwide, as well as other factors like global pollution, the risk of animal-borne diseases, the rise in the number of people who are allergic to milk protein and are lactose intolerant, adopt a flexitarian, vegetarian, or a vegan diet, the interest in plant-based milk and dairy products is continuously increasing. The substantial effect of plant-based products on sustainability, in particular, creates a strong perception and awareness of the consumption of these products. Environmental factors and sustainability are emphasized as the most important reasons for plant-based product consumption because milk of animal origin has a significantly higher environmental effect than all plant-based milk analyzed (Shori and Al Zahrani 2021).

Legumes and grains provide new perspectives in terms of increased nutritional value and adequate protein content. These are affordable foods with high protein and calorie contents. Lupine has early satiety, energizing, lowering the glycemic index (Hall et al. 2005), it regenerates blood lipids (Martins et al. 2005), lowers hypertension (Pilvi et al. 2006), and provides gastrointestinal health benefits (Johnson et al. 2006). Almonds, due to their high protein content, have been shown to reduce the risk of heart disease upon consumption in the daily diet (Hu and Stampfer 1999). Regular consumption of protein-based foods reduces blood pressure (Obarzanek et al. 2001), and low-density lipids and triglycerol concentrations are reduced (Wolfe and Piche 1999). Almond milk is the most commonly used plant-based dairy product in the preparation of mix (Kot et al. 2021). Hazelnut is suitable for diabetics because it has a low glycemic index, and it contains phytochemicals, dietary fibers, and carbohydrates (Bolling et al. 2011). The high vitamin E content in hazelnut lipid profiles, which are primarily oleic acid, lowers cholesterol and reducing the risk of cardiovascular diseases (Tey et al. 2011).

Frozen dessert has a high nutritional value due to its carbohydrates, proteins, and vitamins. Today, the frozen dessert industry has also adapted to consumer demands, and frozen dessert is made from new products other than animal-originated milk, paving way to a new sector, plant-based product market. Nevertheless, if plant-based milk is preferred to eliminate or reduce animal products from our diet, it should not be obtained from a single source. Consumption diversity should be increased to ensure a well-balanced nutrient consumption. As a result, the market for products labeled "blend," which contain more than one source, is expanding (Pontonio et al. 2022). Many studies on frozen dessert have brought new approaches, with researchers attempting to add various functional properties. This functionality, on the other hand, has taken on a new aspect with the employment of pure cultures, which have numerous advantages in dairy technology such standardization of the products (Leahu et al. 2022).

Probiotics and/or prebiotics added to foods improve the functionality of the product. Furthermore, the addition of fruit or protein-rich ingredients to frozen dessert transforms it into a functional product, as do plant-based kinds of milk such as coconut or soy milk. The nutritional content of lupine milk and peanut milk can help lactic acid bacteria grow and survive in frozen dessert, increasing the nutritional components and health benefits of probiotic ice creams (Craig and Brothers 2022).

In the present study, frozen desserts were produced with plant-based milk (almond, hazelnut, and lupine milk) with the addition of *Lb. acidophilus* probiotic culture to increase the functional properties of the product in plant-based milk substitutes. The aim was to determine the quality characteristics of frozen desserts during the 90 day storage and to evaluate efficacy of probiotics in plant-based milk substitutes.

## Materials and methods

### Materials

Sugar, sahlep, condensed rice milk, and milk powder (Pınar Dairy Co. Izmir, Turkey) were supplied from the local market (Izmir, Turkey). The frozen dessert was produced at the Dairy Technology Department Pilot Plant at Ege University Faculty of Agriculture Department of Dairy Technology. *Lb. acidophilus* starter culture was obtained from Chr.Hansen (Chr.Hansen, Istanbul, Turkey) and used in the study in freeze-dried form. The almond and hazelnut milk was supplied from the Fomilk Co. http://www.fomilk.com/en/aboutus.

### Methods

#### Plant-based milk production (almond, hazelnut, lupine)

Lupine milk was obtained by the Sudan method described by Elsamania and Ahmed (2014) using lupine seeds, with slight modifications. Lupine seeds were first exposed to a short-term heat treatment to separate them from their shells. The seeds were kept in 0.06 M NaHCO_3_ aqueous solution for 16 h after being separated from their shells using heat and water. This process aimed to remove the seed's distinct bitterness. After 16 h, the filtered seeds were rinsed twice with water and heat-treated at 100 °C for 20 min to prevent cotyledon formation and stop inter-seed activity. Lupine milk was obtained after shredding in a Soy Quick soy milk extraction machine. All milk samples were cooled and stored at 4 °C in plastic bottles.

The prepared seven types of mixes were inoculated with 4% *Lb. acidophilus* (pH 5.8) in a reconstituted milk prepared from skimmed milk powder. Almond, hazelnut, and lupine mixture combinations comprised almond-hazelnut (v:v), hazelnut-lupine (v:v), almond-lupine (v:v), almond-lupine (v:v) and almond-hazelnut-lupine (v:v:v).

#### Mixtures and frozen dessert production

The procedure of the frozen dessert manufacturing was the addition of plant-based milk for the production of mixtures, homogenization, sahlep and milk concentrate addition, pasteurization, cooling, ripening, and culture addition (*Lb. acidophilus* activated in 25 mL skimmed milk for 30 min). Two batches (replicates) of each frozen dessert samples were made.

#### Physicochemical analyses

The pH, total solids, protein, and fat contents were determined according the AOAC methods (AOAC 2012). Lactose/sucrose/D-glucose contents were determined using Megazyme enzyme kits (K-Lacsu 08/18). The kit’s aim was to reduce the sugars in the medium using enzymes and obtain the three sugars mentioned. Accordingly, a 0.5 g of the sample was weighed into a 50 mL falcon tube and 5 mL of 95% (v:v) ethanol was added. Incubating the solution at 85 °C for 5 min, a 50 mM sodium acetate buffer solution was added to adjust the pH to 4.5. Then, a 1 mL of the filtered filtrate was taken, 3 mL of distilled water was added, and the homogenized mixture was used as the analysis sample. For the analysis, 0.2 mL of the sample was taken and incubated with reducing enzymes for 20 min in a 50 °C water bath. Following the incubation, the sample was re-incubated under same conditions with the glucose oxidase/peroxidase reagent. A spectrophotometric reading was carried out at 510 nm using the lactose/sucrose/D-glucose enzymatic assay procedure (K-LACSU lot 190220–1, Megazyme International).

## Rheological analysis

The viscosity was determined using a Brookfield DV-II Ultra Programmable Rheometer with spindle number 63 at 30 rpm rotation speed. Centi Poise (cP) measurements were carried out at 4 °C.

### Melting test

The frozen dessert’s first dripping and complete melting durations were calculated. Accordingly, 25 g samples were weighed and placed on a room-temperature beaker, and the durations for first dripping and melted complete melting were recorded in min. The melting ratio was calculated by weighing the melted portion after 15, 30, and 45 min. The melting rate was calculated as the function of time, and expressed in g.min^−1^.

### Total phenolic contents

The Folin-Ciocalteu colorimetric method, described by Singleton et al. (1999) was used to determine the total phenolic content of the mixtures and frozen desserts. The absorbance of the samples (at 760 nm wavelength) was measured using a UV–Vis spectrophotometer (Genesys 10 S model, Thermo Scientific, Waltham, MA, USA). Gallic acid was employed as the standard, and the results were expressed as mg of gallic acid equivalent (GAE) per 100 mL of samples (y = 5.5629x + 0.249 R2 = 0.9753).

### Determination of antioxidant capacity by DPPH (2,2-diphenyl-2-picrylhydrazyl)

The radical scavenging activity of the frozen dessert samples was determined as described by Singh et al. (2002). Different concentrations of the mixtures and frozen dessert samples and BHA (25, 50, and 100 ppm) were taken in different test tubes. The volume was adjusted to 100 µL by adding MeOH. Then, a 5.0 mL of 0.1 mM methanolic solution of DPPH was added to these tubes and agitated vigorously. The tubes were allowed to stand at 30 °C for 20 min. The control was prepared without any extract and MeOH was used for the baseline correction. The changes in the absorbance of the samples were measured at 517 nm (Shimadzu UV-1800, Japan). Radical scavenging activity was expressed as the inhibition percentage and was calculated using the following formula;

radical scavenging activity % = (Control OD = sample OD/Control OD) × 100.

The results were expressed as μM Trolox equivalents (TE) per gram of sample (calibration curve linearity range: R^2^ = 0.999).

### Microbiological analyses

The mixtures and frozen dessert samples were diluted and plated using MRS Agar (Merck) for 48 h at 42 °C to count *Lactobacillus* spp*.* and the enumerations were expressed as log CFU/mL. The *Lb. acidophilus counts* were determined using MRS agar medium with the addition of D-sorbitol for 72 h at 37 °C.

### Color

The color of frozen dessert samples was determined using a Minolta Chroma Meter (CR-400; Konica Minolta, Inc.). A2° standard observers function, and illuminant D65 was used as the light source. The CIE L* (lightness), a* (red-green), and b* (yellow-blue) coordinates in the color space were recorded using a white standard calibration plate (Y = 92.7, x = 0.3160, and y = 0.3321). Five measurements were recorded on each sample using the glass light projection tube (Minolta, CR-A33e). The Experiments were carried out as duplicate.

### Sensory evaluation

The samples were stored at − 18 °C for 90 days and the sensory evaluations were carried out by 6 trained panelists who were consumers of frozen dessert (Academic staff and graduate students of the Ege University Dairy Technology Department; four women and two men, aged 28–40) at days 0 and 90. The tests were carried out in lab. About 20 g of samples were served in plastic cups coded with three-digit numbers at a temperature of − 10 °C ± 2 °C, along with a transparent plastic spoon. Between taste of frozen dessert samples, distilled water were provided to refresh the palate. The sensory parameters were color, flavor, taste, texture, and overall acceptability on 5-point hedonic scale (ranging from 1 = ’extremely disliked’ to 5 = ‘extremely liked’) (da Silva et al. 2021).

### Statistical analyses

The SPSS software (SPSS version 21.0, Chicago, IL, USA) was used to analyze the data. The measurements were done in two replications; triplicate tests were conducted for microbiological content and acidity. One-way analysis of variance (one-way ANOVA) was performed for multiple comparisons. The mean values were compared by the Duncan multiple range analysis (*p* < 0.05).

## Results and discussion

Milk quality is an important factor in frozen desserts production. In the present study, the fat content in almond, hazelnut, and lupine milks used as raw materials were 2.30, 2.30, and 1.58%, respectively. The protein contents of the almond, hazelnut, and lupine milks were 1.30, 1.80, and 3.88%, respectively Table 1S). No sugar was detected in lupine milk whereas the sugar content was 2.50% in hazelnut milk and 1.90% in almond milk. The high sugar content of hazelnut milk suggested that the frozen dessert mixtures in the present study may have high sugar values in hazelnut frozen dessert samples. Similarly, lupine milk was determined to have a high protein content and (Asres et al. 2022) determined that plant or fruit-based milk addition, such as peanut milk or lupine milk, can affect the physical properties of frozen desserts.

**Table 1:** Some physicochemical parameters of frozen dessert mixes

	AM	HM	LM	AHM	HLM	ALM	AHLM
pH	6.50 ± 0.00^a^	6.61 ± 0.00^a^	6.38 ± 0.00^a^	6.46 ± 0.00^a^	6.55 ± 0.00^a^	6..45 ± 0.00^a^	6..48 ± 0.00^a^
Dry matter (%)	31.35 ± 0..04^d^	32.43 ± 0..12^ cd^	38.53 ± 0..5^a^	32.53 ± 0.06^bc^	35.21 ± 0.61^b^	34..60 ± 0.22^b^	34..35 ± 0.56^bc^
Protein (%)	5.47 ± 0.0^c^	4.32 ± 0.2^d^	7.77 ± 0.06^a^	4..9 ± 0.03^ cd^	6.08 ± 0.04^b^	6.29 ± 0..04^b^	5..40 ± 0.03^c^

Values are means ± standard deviations of three independent trials

^a,b,c,d^ Represent storage days (0–90) values with the same lower case letters in the same row do not differ significantly (*p* > 0.05)

^A,B,C,D,E^ Represent within same storage days values with the same upper case letters in the same column do not differ significantly (*p* > 0.05)

*AM* almond milk, *HM* hazelnut milk, *LM* lupine milk, *AHM* almond-hazelnut milk, *HLM* hazelnut-lupine milk, *ALM* almond-lupine milk, *AHLM* almond-hazelnut-lupine milk

In addition to plant-based milk, the mixtures contained sugar, milk powder, sahlep, and condensed rice milk. The pH of the mixtures ranged from 6.45 to 6.60 (*p* > 0.05). The dry matter values ranged from 31.35 to 38.53% (*p* < 0.05). The formation of the non-homogenized particulate structure during lupine milk production resulted in an increase in the dry matter values (Table 1). Szydłowska and Kołożyn-Krajewska (2019) reported that the dry matter values are about 24% in the probiotic and symbiotic pumpkin frozen desserts. The Lb. *acidophilus* count was determined to be 9.02–10.84 log CFU/g (*p* < 0.05). The probiotic bacteria content of almond milk was associated with sugar utilization of *Lb*. *acidophilus*. The high levels of *Lb. acidophilus* in the mixes in the present study compared to other studies showed that vegetable sugars contribute positively to *Lb. acidophilus* counts.

The prepared frozen dessert mixtures were heated before adding milk powder and sugar, homogenization, sahlep, and rice milk concentrate. Table 2 shows the physicochemical properties of frozen dessert samples obtained through pasteurization, cooling, ripening, and culture addition, and production in the batch system. The dry matter contents determined on the first day after the production of frozen dessert samples ranged from 30.56 to 37.10% (*p* < 0.05). The frozen desserts containing lupine milk were thought to had the highest ratio and other following high values, associated with the particle structure originating from lupine milk production. The results were comparable to those of other studies on ice-cream/frozen dessert containing plant-based milk (Amirtha et al. 2021). The differences in fat contents was associated with the fat content of the plants, particularly in the case of hazelnut milk frozen dessert fat. The fat content of soy milk frozen dessert was 7.47%, which was higher than the fat content of lupine milk frozen dessert (6.6%). The frozen dessert samples containing lupine milk had the highest protein content (8.1%) and the samples containing hazelnut milk had 4.38% protein content, and the differences were found to be significant (*p* < 0.05). Protein contributes to the air incorporation into the mixture, which produces small air bubbles and the texture in the ice cream. It also helps with the emulsification of the fat by maintaining the suspension of the fat molecules in the mixture. In contrast to this study, the protein content of frozen desserts containing sweet beans and soy milk ranged from 4.27 to 4.79% (Asres et al. 2022). The D-glucose values were examined, the hazelnut milk sample had the highest value whereas the hazelnut-lupine milk sample had the lowest. The D-glucose value, which indicates the degree of rotation of polarized light, reached its maximum in the hazelnut milk samples. The hazelnut-lupine milk samples had the lowest value (Table 2). The lupine milk frozen dessert had the highest sucrose ratio at 6.64% whereas almond milk samples had the lowest at 4.60% (*p* < 0.05). It is assumed that the greater amount of sucrose in lupine milk may be due to the fact that some of the indigestible sugars in the composition of lupine become soluble, particularly after soaking to partially reduce bitterness. It is hypothesized that the greater amount of sucrose in lupine milk was due to some of the indigestible sugars in the lupine component being soluble, particularly after soaking to partially reduce bitterness. Table 1 shows the acidity of the frozen dessert samples ranged from 6.38 to 6.61 (*p* > 0.05). The pH values of frozen dessert produced with different proportions of soy milk ranged from 6.44 to 7.33 (Ng et al. 2023).

**Table 2:** Some physicochemical parameters of plant-based frozen desserts

%	AM	HM	LM	AHM	HLM	ALM	AHLM
Dry matter	30.56 ± 0.011^E^	31.39 ± 0.21^D^	37.10 ± 0.05^A^	31.52 ± 0.06^D^	33.89 ± 0.09^C^	34.60 ± 0.23^B^	33.89 ± 0.09^C^
Fat	1.74 ± 0.00^CD^	2.73 ± 0.29^A^	1.22 ± 0.03^E^	2.31 ± 0.00^B^	1.91 ± 0.05^C^	1.55 ± 0.04^D^	1.75 ± 0.00^CD^
Protein	4.93 ± 0.03^E^	4.38 ± 0.02^G^	8.10 ± 0.06^A^	4.7600.05^F^	6.26 ± 0.03^C^	6.63 ± 0.04^B^	5.92 ± 0.09^D^
Ash	1.27 ± 0.013^AB^	1.30 ± 0.014^A^	1.18 ± 0.08^BC^	1.17 ± 0.032^BC^	1.36 ± 0.03^A^	1.28 ± 0.04^AB^	1.14 ± 0.01^C^
D-glucose	58.57 ± 4.57^B^	78.70 ± 3.85^A^	63.17 ± 1.15^B^	49.3 ± 5.02^CD^	47.34 ± 4.83^D^	55.17 ± 7.81^C^	77.54 ± 7.08^A^
Sucrose	5.85 ± 0.51^B^	4.68 ± 0.06^DE^	6.64 ± 0.15^A^	4.82 ± 0.11^D^	5.35 ± 0.09^C^	4.60 ± 0.08^E^	5.89 ± 0.11^B^
Lactose	7.32 ± 0.03^D^	7.41 ± 0.02^BC^	7.48 ± 0.02^A^	7.42 ± 0.02^BC^	7.47 ± 0.05^A^	7.45 ± 0.03^AB^	7.39 ± 0.03^C^

Values are means ± standard deviations of three independent trials

^a,b,c,d^ Represent storage days (0–90) values with the same lower case letters in the same row do not differ significantly (*p* > 0.05)

^A,B,C,D,E^ Represent within same storage days values with the same upper case letters in the same column do not differ significantly (*p* > 0.05)

*AM* almond milk, *HM* hazelnut milk, *LM* lupine milk, *AHM* almond-hazelnut milk, *HLM* hazelnut-lupine milk, *ALM* almond-lupine milk, *AHLM* almond-hazelnut-lupine milk

Due to the relationship with free radical-related diseases, LDL lipid peroxidation, and atherosclerosis, the role of phenolic compounds in plant foods has become significant in recent years. Table 3 shows the antioxidant capacity determined in frozen dessert samples on day 0 and 90. Almond-hazelnut-lupine milk frozen dessert had the lowest value (18.83 mg GAE g^−1^ on day 0), whereas lupine milk had the highest value of 75.06 mg GAE g^−1^ (*p* < 0.05). The samples from the first day of storage had the highest and lowest values of total phenolic compounds (TPC). The TPC value in quinoa milk probiotic dessert ranged from 2.75–17.58 mg GAE kg^−1^ (Yarabbi et al. 2023). TPC values were affected by the type of plant-based milk used, the production parameters, the heat treatment and storage methods used during the mix's manufacture, and the oxidation-like reactions that occurred during the preparation of mixes, and the oxidation-like reactions that occurred in this process. Genetic factors, environmental factors, and process parameters all have an effect the phenolic compound values. Natural antioxidants work by neutralizing harmful free radicals in the human body and preserving fat-rich foods. The samples with the highest antioxidant capacity were found on day 90 day in lupine frozen dessert whereas the samples with the lowest antioxidant capacity were found on day 0 in the almond-hazelnut-lupine frozen dessert (*p* < 0.05). Ice creams and frozen desserts containing plant-based milk promoted the development of probiotic microorganisms in these samples due to the high phenolic components and antioxidant capacity. The lupine milk frozen dessert had the highest DPPH values (87.28), whereas the almond-hazelnut-lupine milk frozen dessert frozen had the lowest DPPH values (38.28) on day 0 (*p* < 0.05). These results indicated the possible interaction of plant phenolics with microbial proteins that can form insoluble complexes and could reduce antioxidant capacity of samples. The antioxidant capacity values of the frozen desserts prepared using water-soluble extract of rice by-product and Spirulina *Platensis* during storage (days 1 and 120) were reported to be in the range of 5.0–18% (de Souza et al. 2023).

**Table 3:** Total phenolic content and antioxidant activity of plant-based frozen desserts

	TPC ((mg GAE g^−1^)		DPPH (mM)	
	day 0	day 90	day 0	day 90
AM	30.61 ± 0.93^d^	26.79 ± 1.42^e^	60.50 ± 0.00^b^	55.49 ± 0.004^f^
HM	42.30 ± 0.67^c^	44.65 ± 0.78^c^	43.77 ± 0.004^e^	72.15 ± 0.001^d^
LM	75.06 ± 0.65^a^	57.14 ± 2.71^a^	43.45 ± 0.001^f^	87.28 ± 0.007^a^
AHM	25.97 ± 6.87^de^	38.05 ± 2.00^d^	58.01 ± 0.003^d^	58.71 ± 0.003^e^
HLM	55.73 ± 0.03^b^	50.02 ± 0.40^b^	59.59 ± 0.000^c^	84.41 ± 0.006^b^
ALM	53.34 ± 0.65^b^	45.11 ± 2.95^c^	67.86 ± 0.000^a^	77.65 ± 0.000^c^
AHLM	18.83 ± 4.56^e^	27.55 ± 1.71^e^	38.28 ± 0.006^ g^	46.21 ± 0.005^ g^

Values are means ± standard deviations of three independent trials

^a,b,c,d^ Represent storage days (0–90) values with the same lower case letters in the same row do not differ significantly (*p* > 0.05)

^A,B,C,D,E^ Represent within same storage days values with the same upper case letters in the same column do not differ significantly (*p* > 0.05)

*AM* almond milk, *HM* hazelnut milk, *LM* lupine milk, *AHM* almond-hazelnut milk, *HLM* hazelnut-lupine milk, *ALM* almond-lupine milk, *AHLM* almond-hazelnut-lupine milk

*Lb. acidophilus* starter culture (Chr. Hansen) was used in the study for its probiotic properties (Table 4). During the 90 day-storage, *Lb. acidophilus* ranged from 3.23 to 8.99 CFU/mL (*p* < 0.05). The almond-lupine milk frozen dessert had the highest count on the first day of storage, whereas the lupin milk frozen dessert had the lowest count on day 60 of storage (*p* < 0.05). The increased count of *Lb. acidophilus* in lupine milk frozen dessert was attributed to the higher concentration of dietary fiber in lupine's composition (~ 28%) (Asres et al. 2022). *Lb. acidophilus* count ranged from 5.26–8.20 log CFU/g on frozen dessert prepared using various combinations of coconut or bovine milks with soya milk (Aboulfazlı et al. 2016).

**Table 4:** *Lb. acidophilus* viability of plant-based frozen desserts (log CFU/mL)

	day 0	day 30	day 60	day 90
AM	5.41 ± 1.14^BCa^	5.81 ± 0.06^BCa^	3.87 ± 1.19^CDa^	6.04 ± 0.81^ABa^
HM	6.19 ± 0.26^BCa^	5.81 ± 0.94^BCa^	3.22 ± 2.33^Db^	4.09 ± 0.58^Cab^
LM	6.69 ± 0.24^Ba^	4.75 ± 0.51^Cb^	5.33 ± 0.13^ABCab^	3.61 ± 2.07^Cb^
AHM	8.99 ± 0.45^Aa^	8.08 ± 0.01^Ab^	6.47 ± 0.59^Ac^	7.06 ± 0.06^Ac^
HLM	8.85 ± 0.44^Aa^	6.69 ± 0.44^Bb^	5.98 ± 0.15^ABc^	6.29 ± 0.16^ABbc^
ALM	4.15 ± 2.21^Ca^	4.84 ± 0.18^Ca^	4.30 ± 1.13^BCDa^	5.38 ± 0.22^ABCa^
AHLM	6.12 ± 0.71^BCa^	5.03 ± 1.23^Ca^	6.11 ± 0.05^ABa^	4.68 ± 1.13^BCa^

Values are means ± standard deviations of three independent trials

^a,b,c,d^ Represent storage days (0–90) values with the same lower case letters in the same row do not differ significantly (*p* > 0.05)

^A,B,C,D,E^ Represent within same storage days values with the same upper case letters in the same column do not differ significantly (*p* > 0.05)

*AM* almond milk, *HM* hazelnut milk, *LM* lupine milk, *AHM* almond-hazelnut milk, *HLM* hazelnut-lupine milk, *ALM* almond-lupine milk, *AHLM* almond-hazelnut-lupine milk

The microbial content must be able to maintain its viability to maintain the probiotic properties of the products produced. *Lb. acidophilus* counts indicate that the plant-based milk samples produced retained its probiotic properties. *Lb. plantarum* viability was preserved after the 120 day storage period, lactic acid bacterial counts in plant-based ice creams containing *Lb. brevis* was found to be higher than 10^7^ CFU/mL (Pontonio et al. 2022).

It is critical for food quality to monitor color changes during storage, ripening, and processing. Table 5 displays the color values of plant-based probiotic frozen dessert samples. The L* value is the light and dark criterion in the Hunter color scale, and was determined to be 73.59 on day 90 of the hazelnut samples, and the almond milk frozen dessert sample had the lightest color with 86.95 on the first-day measurements. The closeness of the samples to green color is shown by Hunter color evaluation. The difference of color parameters in all samples could be explained by the effect of pigments such as carotenoids, and flavonoids, which are responsible for the red, yellow, and orange colors in dairy and plant based products such as fortified plant milks.

**Table 5:** Color values of plant-based frozen desserts

	L (Lightness)				a (Redness)				B (yellowness)			
	0th day	30th day	60th day	90th day	0th day	30th day	60th day	90th day	0th day	30th day	60th day	90th day
AM	86.95 ± 0.14^Aa^	86.37 ± 0.71^Aa^	85.31 ± 0.13^Ab^	86.52 ± 0.31^Aa^	− 6.74 ± 0.05^DEb^	− 6.69 ± 0.08^Db^	− 6.50 ± 0.02^ Da^	− 7.08 ± 0.06^Fc^	14.35 ± 0.21^ Da^	14.44 ± 0.90^Ca^	14.02 ± 0.18^Eab^	13.18 ± 0.66^EFb^
HM	75.32 ± 0.49^Ca^	74.25 ± 0.34^Fb^	73.96 ± 0.021^Cb^	73.59 ± 0.075^Eb^	− 0.62 ± 0.12^Ac^	− 0.14 ± 0.06^Aa^	− 0.08 ± 0.05^Aa^	− 0.33 ± 0.03^Ab^	14.09 ± 0.59^Dab^	14.72 ± 0.14^Ca^	14.32 ± 0.00^DEab^	13.55 ± 0.43^Eb^
LM	84.37 ± 0.39^ABb^	84.39 ± 0.29^Bb^	84.99 ± 0.176^Aa^	84.42 ± 0.075^Bab^	− 6.64 ± 0.08^ Da^	− 6.67 ± 0.05^ Da^	− 6.59 ± 0.04^Ea^	− 6.55 ± 0.02^Ea^	22.85 ± 0.66^Aa^	22.70 ± 0.46^Aab^	22.15 ± 0.00^Aab^	21.85 ± 0.08^Ab^
AHM	76.81 ± 0.52^Cc^	75.71 ± 1.78^Ec^	79.03 ± 0.133^Bb^	84.02 ± 0.19^Ba^	− 2.75 ± 0.18^Bb^	− 2.30 ± 0.07^Ba^	− 2.37 ± 0.03^Ba^	− 2.23 ± ± 0.05^Ca^	14.05 ± 0.52^ Da^	14.24 ± 0.44^Ca^	12.77 ± 0.15^Fb^	12.63 ± 0.09^Fb^
HLM	78.73 ± 0.57^BCa^	77.87 ± 1.12^ Da^	78.66 ± 0.190^Ba^	78.14 ± 0.19^ Da^	− 2.83 ± 0.03^Bc^	− 2.47 ± 0.12^Bb^	− 2.44 ± 0.05^Bb^	− 2.03 ± 0.02^Ba^	16.57 ± 0.28^Ca^	15.23 ± 0.43^Cb^	14.79 ± 0.07^CDb^	17.52 ± 0.77^Ba^
ALM	79.86 ± 9.93^BCa^	85.04 ± 0.076^Ba^	84.66 ± 0.20^Aa^	82.40 ± 1.00^Ca^	− 6.84 ± 0.16^Eb^	− 6.78 ± 0.02^Dab^	− 6.61 ± 0.02^Ea^	− 7.275 ± 0.06^Gc^	20.68 ± 1.23^Ba^	18.66 ± 0.04^Bb^	18.15 ± 0.65^Bbc^	16.68 ± 0.30^Cc^
AHLM	81.24 ± 0.92^ABCab^	80.74 ± 0.23^Cbc^	79.59 ± 1.16^Bc^	82.40 ± 0.72^Ca^	− 3.64 ± 0.07^Cb^	− 3.60 ± 0.13^Cb^	− 3.18 ± 0.07^Ca^	− 4.35 ± 0.02^Dc^	16.19 ± 0.25^Cb^	16.90 ± 2.99^Ca^	15.09 ± 0.13^Cc^	15.12 ± 0.44^Dc^

Values are means ± standard deviations of three independent trials

^a,b,c,d^ Represent storage days (0–90) values with the same lower case letters in the same row do not differ significantly (*p* > 0.05)

^A,B,C,D,E^ Represent within same storage days values with the same upper case letters in the same column do not differ significantly (*p* > 0.05)

*AM* almond milk, *HM* hazelnut milk, *LM* lupine milk, *AHM* almond-hazelnut milk, *HLM* hazelnut-lupine milk, *ALM* almond-lupine milk, *AHLM* almond-hazelnut-lupine milk

**Fig. 1:**
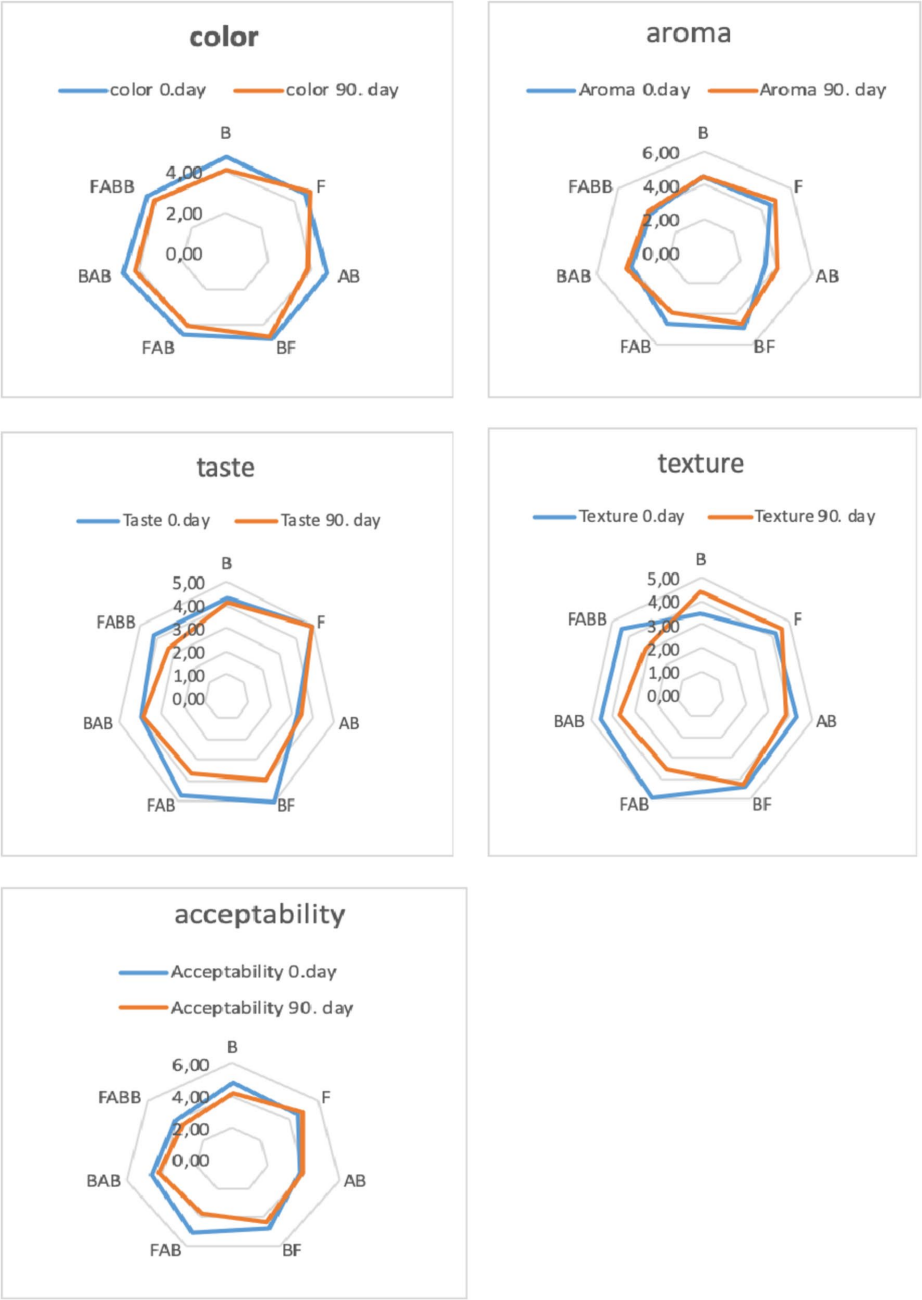
Sensory scores of frozen dessert samples

Frozen desserts were evaluated by a trained panelist team for color, aroma, taste, texture, and overall acceptability (Fig. 1). According to the sensory analysis results, color evaluations ranged from 3.75 to 5.0, with the hazelnut milk frozen sample receiving the highest scores. The color parameters of the almond-hazelnut milk frozen dessert samples had the highest sensory panelists. The aroma scores of the samples ranged from 3.40 to 5.0 (*p* < 0.05). The hazelnut milk frozen dessert sample had the highest aroma score, whereas the lupine milk frozen dessert sample had the lowest. Although the bitter smell from the lupine raw material was reduced with sodium bicarbonate, it could not be completely eliminated and therefore received low scores in the sensory evaluation. Similarly, the panelists appreciated the hazelnut aroma, which resulted in the highest average score in sensory evaluation (Fig. 1). The texture scores in the plant-based probiotic frozen dessert samples ranged from 3.1 to 5.0, with the almond milk frozen dessert sample having the lowest texture scores whereas the hazelnut milk sample had the highest texture. The hazelnut milk frozen dessert received the highest overall acceptability scores. The lupine milk frozen dessert received the lowest overall acceptability scores. In terms of overall taste, the sandy structure left in the mouth by the lupine milk frozen dessert resulted in the lowest scores since the overall bitterness was at a considerable level even when reduced. In terms of frozen dessert characteristics such as appearance, mouth feel, and odor intensity, the hazelnut milk frozen dessert had the highest scores. Da Silva et al. (2021) have reported that a vegan and nonvegan consumers have a similar perception about prebiotic frozen dessert processed with water-soluble extract of rice byproduct. The sensory qualities of frozen desserts samples were assessed which made with non-dairy components and various probiotic cultures added. The addition of probiotic cultures usually does not alter the sensory properties (flavor, color, texture, and overall acceptance) of the products (Pimentel et al. 2021). However, the type of raw material (vegetable extract), probiotic culture, and type of frozen dessert (fermented or not) may have an impact on the sensory perception of the products.

One of the most important parameters that have a role in evaluating the quality of frozen desserts is the viscosity which contributes to the desired body and texture of products. Therefore, the measurement of viscosity is important to know the effect of the addition of plant-based milk on the frozen dessert attributes. As shown in Table 6S, the changes in the viscosity values of the plant-based probiotic frozen dessert samples were found to be significant (*p* < 0.05) both during the storage period and between the samples. The lupine milk frozen dessert samples had the highest viscosity, whereas the almond milk frozen desserts had the lowest value on the first day of storage (*p* < 0.05). The viscosity values of almond-hazelnut milk, hazelnut-lupine milk, and the almond-hazelnut-lupine milk frozen dessert samples were close to each other on day 90 day of storage. The higher viscosity value in the lupine milk frozen dessert sample compared to other frozen dessert samples was associated with the particle structure in raw material production. These findings could be attributed to plant-based milk’s high fiber content, which holds extra water, increasing the viscosity of the final product (El-Said et al. 2021). It was stated that the high protein content and the binding capacity of the proteins due to their interaction with water increased the viscosity of the mix (Onuorah et al. 2007). The longest and shortest dripping times were observed on day 30 of storage when the first dripping times of the frozen dessert samples were examined (*p* < 0.05). The lupine milk frozen dessert sample drips for the first time after 26 min and had the longest first dripping time, whereas the almond-hazelnut-lupine milk sample drips after 5 min and had the shortest first dripping time. For the frozen dessert industry, the first dripping time is an important feature for temperature resistance. Considering the first dripping times according to storage days, the first dripping times in almond milk and lupine milk frozen dessert samples were longer than 16 min, these two frozen desserts had greater heat resistance than other samples. Bilbao‐Sainz et al. (2019) showed that addition of strawberry, raspberry, or blackberry powder completely prevented the melting of the frozen desserts. Also they observed a significant reduction in the melting rates of the samples (20–30 min).

The higher protein content in higher hazelnut milk-contained samples can slow down melting rates due to their foam-forming capacity of proteins and higher viscosity. In the present study, there were significant differences (*p* < 0.05) between the storage days of the frozen dessert samples when they were completely melted and between the samples belonging to the same storage day. The duration to completely melt on day in almond milk frozen sample was the longest (69 min), and the hazelnut-lupine milk frozen desert melted in the shortest duration (41 min) compared to other samples (Table 6S). Ice cream and frozen desserts are multiphase systems composed of ice crystals, air cells, partially-fat clusters, fat globules, and an unfrozen serum phase. The melting resistance ranged from 83.01 to 84.50% for soymilk, for cow milk ice cream samples and from 83.23 to 84.40% for sweet lupine milk-cow milk ice cream samples at different blend ratios. As the amount of soy and sweet lupine milk used in ice cream production increased, the melting resistance increased (Asres et al. 2022).

## Conclusion

In the present study, using plant-based milk had no negative effects on the physicochemical properties of frozen desserts and caused significant differences in various properties. Although the general properties of the frozen desserts produced in the present study were similar, no similar properties were found in distinguishing properties such as viscosity and melting duration. Different ingredients thought to be capable of standardizing the frozen dessert production process and plant-based milk compositions utilized in the production of high-quality, as well as supporting attributes such as flavor. Probiotic microorganisms are added to new functional frozen desserts produced by three different plant-based milks providing health benefits. Frozen desserts containing plant-based milk stimulated the development of probiotic microorganisms in these samples due to the high phenolic components and antioxidant capacity. Consumer demand has given rise to a functional product market, a new market in which the orientation toward plant-based milk products is rapidly increasing and the use of plant materials in frozen dessert production have become more common. In conclusion, the sensory scores were related with the commercialization of hazelnut milk utilized in plant-based frozen dessert such as ice cream production. It could be marketed as having healthy benefits and being a consumable product.

## Data and code availability

The datasets used and/or analyzed during the current study are available from the corresponding author on reasonable request.

## Supplementary Information

Below is the link to the electronic supplementary material.

Supplementary file 1 (DOCX 23 KB)
