# Supplementary material for: Manufacturing plant-based non-dairy and probiotic frozen desserts and their impact on physicochemical, sensory and functional aspects
Source: J Food Sci Technol. 2024 Mar 10;61(10):1874–83. doi: 10.1007/s13197-024-05964-8 (PMC11401809; doi:10.1007/s13197-024-05964-8)
Supplement: Supplementary file 1 — (DOCX 23 KB) [file 13197_2024_5964_MOESM1_ESM.docx]

**Supplementary Tables**

**Table 1S.** Components of plant based milks

| **%** | **Fat** | **CHO** | **Total Sugar** | **Protein** |
| --- | --- | --- | --- | --- |
| Almond milk | 2.3 | 4.4 | 1.9 | 1.3 |
| Hazelnut milk | 2.3 | 4.0 | 2.5 | 1.8 |
| Lupine milk | 1.6 | - | - | 3.9 |

Values are means ± standard deviations of three independent trials.

**Table 6S**. Viscosity and melting times of plant-based frozen desserts (cP).

|  | **viscosity (cP)** | | | | **First dropping melting times (min)** | | | | **Complete melting times (min)** | | | | |
| --- | --- | --- | --- | --- | --- | --- | --- | --- | --- | --- | --- | --- | --- |
|  | **0^th^ day** | **30^th^ day** | **60^th^ day** | **90^th^ day** | **0^th^ day** | **30^th^ day** | **60^th^ day** | **90^th^ day** | **0^th^ day** | **30^th^ day** | **60^th^ day** | **90^th^ day** |  |
| **AM** | 54±11.53^Ea^ | 64±0.70^Ea^ | 58±0.70^Ea^ | 65±0.70^Da^ | 18±0.00^Bcd^ | 24±0.00^Ab^ | 11.67±0.00^Ca^ | 12±0.00^Ca^ | 69.00±0.00^Aa^ | 63.00±0.00^Ab^ | 35.00±0.00^Cd^ | 45.00±0.00^Bb^ |  |
| **HM** | 231±4.94^Ca^ | 236±0.70^Ca^ | 240±18.38^Ca^ | 193±7.07^Cb^ | 23±0.00^Aa^ | 24±0.00^Ab^ | 7.67±0.00^Cbc^ | 11.67±0.00^Ba^ | 51.00±0.00^Bb^ | 65.00±0.00^Ab^ | 48.00±0.00^Ca^ | 43.00±0.00^Cc^ |  |
| **LM** | 780±16.97^Ac^ | 783±19.09^Ac^ | 1046±0.70^Ab^ | 1082±0.70^Aa^ | 19±0.00^Bc^ | 26±0.00^Aa^ | 9±0.00^Db^ | 11.33±0.00^Ca^ | 62.00±0.00^Ba^ | 68.00±0.00^Aa^ | 42.00±0.00^Cc^ | 45.00±0.00^Cb^ |  |
| **AHM** | 132±0.70^Dab^ | 121±0.70^Da^ | 139±12.02^Dab^ | 248±84.85^BCa^ | 18±0.00^Bcd^ | 23±0.00^Ab^ | 6±0.00^Dcd^ | 12±0.00^Ca^ | 54.00±0.00^Ab^ | 56.00±0.00^Ac^ | 45.00±0.00^Bb^ | 45.00±0.00^Bb^ |  |
| **HLM** | 277±4.24^Bb^ | 268±7.77^Bb^ | 389±10.60^Ba^ | 286±2.82^BCb^ | 21.33±0.00^Ab^ | 23±0.00^Ab^ | 6±0.00^Ccd^ | 12±0.00^Ba^ | 42.00±0.00^Cc^ | 55.00±0.00^Ac^ | 49.00±0.00^Ba^ | 41.00±0.00^Cc^ |  |
| **ALM** | 240±33.23^Cb^ | 235±5.65^Cb^ | 357±9.89^Ba^ | 315±35.35^Ba^ | 15±0.00^Be^ | 23±0.00^Ab^ | 5±0.00^Dd^ | 9±0.00^Cb^ | 45.00±0.00^Bc^ | 52.00±0.00^Ac^ | 40.00±0.00^Cc^ | 46.00±0.00^Ba^ |  |
| **AHLM** | 149±5.65^Da^ | 146±0.00^Da^ | 230±28.99^Ca^ | 239±84.85^BCa^ | 17±0.00^Ad^ | 5±0.00^Cc^ | 6±0.00^Ccd^ | 8.25±0.00^Bb^ | 45.00±0.00^Ac^ | 43.00±0.00^Bd^ | 45.00±0.00^Ab^ | 45.00±0.00^Ab^ |  |

Values are means ± standard deviations of three independent trials.

^a a,b,c,d^ represent storage days (0-90) values with the same lower case letters in the same row do not differ significantly (p>0.05)

^A,B,C,D,E^ represent within same storage days values with the same upper case letters in the same column do not differ significantly (p>0.05)

AM: Almond milk; HM: Hazelnut milk; LM: Lupine milk; AHM; Almond-hazelnut milk; HLM: Hazelnut-lupine milk; ALM: Almond-lupine milk: AHLM: Almond-hazelnut-lupine milk
